# Contagiousness of Typhoid Fever

**Published:** 1852-07

**Authors:** 


### THE WESTERN JOURNAL.

—

THIRD SERIES.—VOL. X.—NO. I.

—

LOUISVILLE, JULY 1, 1852.

---

### CONTAGIOUSNESS OF TYPHOID EEVER.

Dr. A. B. Chambers, of Warsaw, Kentucky, has communicated to us the following facts bearing upon the *contagiousness of Typhoid Fever*:

"I have a friend residing on a wide bottom of Eagle creek, in this county. He has lived there for ten or fifteen years, his family enjoying excellent health. The latter part of last summer he sent out one of his daughters to school in Louisville, where she remained about two months, when becoming indisposed she was brought home. Her sickness proved to be typhoid fever in a very distinct form. Her recovery, which took took place in from three to five weeks—not now distinctly remembered—was followed by an attack of her brother, and subsequently of her parents, her grandfather, who resided in the family, and all her other brothers and sisters (six in number) except one, and some seven or eight negroes. When all the other members of the white family had become sick, the little daughter, who had as yet escaped, was sent to the house of the gentleman's mother, in the hope that a change of locality and removal from the contaminated atmosphere of home might secure her from an attack. She remained there for three weeks, to all appearance in perfect health, with the exception of a slight inflammation of one of her eyes, which yielded to simple treatment. At the end of this time she was taken down with the same fever, in a well marked form, and was sick for two months. In the latter part of the winter, a young lady, a relative of the family, residing at Midway, in Woodford county, hearing of the affliction of her friends, came down to assist in nursing them. She remained two weeks in constant attendance on several of the family who were then slowly recovering, and then paid a visit to the little daughter who was lying sick. At this house she was attacked in a day or two, and had a hard spell of fever, lasting some six weeks, but is now again restored to health. In the early part of her sickness, a negro girl belonging to another member of the family, was employed to assist in nursing, and particularly to wash the clothes of the sick persons. Some ten or fifteen days after returning home, she was attacked with the disease in a mild form, so mild that it did not confine her constantly to bed, but still with the characteristic symptoms distinctly marked—such as muscular debility, continued fever, rapid pulse, abdominal tenderness, liquid stools, &c. She is now well; but a negro man who slept in the same house with her, has been attacked with the same symptoms in a severe form. In addition to these, a brother of the gentleman, who spent some days in his family during their sickness, on returning to his home, was sick for some two weeks, though of the precise character of his symptoms I am not informed, as I did not see him till he was much improved. He thinks, however, that his disease was similar in character though not so severe as that he saw in his brother's family.

Up to this time there had been but little of this fever in our county, in comparison with many in the interior of the state; and during the period mentioned there was not more among the other inhabitants than usual.

Until those cases came under my observation I had not met with anything to authorise the belief that typhoid fever is contagious, nor did I believe it; but I confess that if my opinion has not undergone an entire change, my faith in its non-contagious character is greatly shaken.

Some of these cases presented the disease in a grave form; in most of them it was mild; but in all the symptoms were highly characteristic."

Warsaw, May 13th, 1852.

